# Epigenetic Inhibitors Differentially Impact TGF-β1 Signaling Cascades in COPD Airway Smooth Muscle Cells

**DOI:** 10.3390/cells14010031

**Published:** 2024-12-31

**Authors:** Karosham Diren Reddy, Dikaia Xenaki, Ian M. Adcock, Brian G. G. Oliver, Razia Zakarya

**Affiliations:** 1 Respiratory Cellular and Molecular Biology Group, Woolcock Institute of Medical Research, Macquarie University, Macquarie Park, Sydney, NSW 2113, Australia; dia.xenaki@woolcock.org.au (D.X.); brian.oliver@uts.edu.au (B.G.G.O.); 2 School of Life Science, University of Technology Sydney, Ultimo, NSW 2007, Australia; 3 Airways Disease, Respiratory Cell & Molecular Biology, Airways Disease Section, National Heart and Lung Institute, Faculty of Medicine, Imperial College London, London SW7 2BX, UK; ian.adcock@imperial.ac.uk; 4 Epigenetics of Chronic Disease Group, Woolcock Institute of Medical Research, Macquarie University, Macquarie Park, Sydney, NSW 2113, Australia

**Keywords:** COPD, epigenetics, airway smooth muscle, inflammation, DNA methylation, histone acetylation, CXCL8, TGF-beta, therapeutics

## Abstract

**Background:** Chronic obstructive pulmonary disease (COPD) is characterized by progressive and incurable airflow obstruction and chronic inflammation. Both TGF-β1 and CXCL8 have been well described as fundamental to COPD progression. DNA methylation and histone acetylation, which are well-understood epigenetic mechanisms regulating gene expression, are associated with COPD progression. However, a deeper understanding of the complex mechanisms associated with DNA methylation, histone post-translational changes and RNA methylation in the context of regulatory pathways remains to be elucidated. We here report on how DNA methylation and histone acetylation inhibition differentially affect CXCL8 signaling in primary human non-COPD and COPD airway cells. **Methods:** Airway smooth muscle (ASM) cells, a pivotal cell type in COPD, were isolated from the small airways of heavy smokers with and without COPD. Histone acetylation and DNA methylation were inhibited before the TGF-β1 stimulation of cells. Subsequently, CXCL8 production and the abundance and activation of pertinent transcription regulatory proteins (NF-κB, p38 MAPK and JNK) were analyzed. **Results:** TGF-β1-stimulated CXCL8 release from ASM cells from ‘healthy’ smoker subjects was significantly modulated by DNA methylation (56.32 pg/mL and 56.60 pg/mL) and acetylation inhibitors (27.50 pg/mL and 48.85 pg/mL) at 24 and 48 h, respectively. However, modulation via the inhibition of DNA methylation (34.06 pg/mL and 43.18 pg/mL) and acetylation (23.14 pg/mL and 27.18 pg/mL) was observed to a lesser extent in COPD ASM cells. These changes were associated with differences in the TGF-β1 activation of NF-κB and MAPK pathways at 10 and 20 min. **Conclusions:** Our findings offer insight into differential epigenetics in controlling COPD ASM cells and provide a foundation warranting future studies on epigenetic differences associated with COPD diagnosis. This would provide a scope for developing therapeutic interventions targeting signaling and epigenetic pathways to improve patient outcomes.

## 1. Introduction

Chronic obstructive pulmonary disease (COPD) is characterized by small airway inflammation leading to chronic bronchitis, followed by airway fibrosis and the destruction of the alveoli and emphysema. These factors culminate in a severe and progressive loss of lung function in the patient. Although treatments for COPD exist, there is no cure for the progressive disease, with the only effective therapeutic intervention being lung transplants. The lumen of the small airways (<2 mm) is narrowed by the thickening of the airway wall in the early stages of the disease [1]. Although many noxious stimuli contribute to COPD pathogenesis, cigarette smoking is the predominant environmental insult causing disease and contributing to accelerated lung function decline. Although most patients with COPD have a significant smoking history, not all smokers develop COPD. Therefore, it is pertinent to delineate the physiological and molecular differences between smokers who do and do not develop COPD.

It is posited that small airway fibrosis in COPD is preceded by early small airway inflammation, leading to extracellular matrix remodeling underpinning airway obstruction. The airway smooth muscle (ASM) presents as an understudied yet pivotal cell type contributing to the progression of COPD [2]. ASM is activated by systemic inflammation, leading to the development of hypertrophy and hyperplasia in COPD [2]. TGF-β is a major pleiotropic cytokine promoting fibrotic and proinflammatory phenotypes in COPD, specifically in ASM cells. The ASM has been long established to contribute to both the inflammatory and remodeling processes in the small airways [3,4].

Epigenetics encompasses mitotically heritable molecular features that alter gene expression without altering the gene sequence. The most well-understood epigenetic marks are DNA methylation and histone acetylation, which occur on DNA and histone tails. DNA methylation is mediated by a class of enzymes known as DNA methyltransferases (DNMTs); of this group of enzymes, DNMT1 plays the most predominant role in maintaining DNA methylation marks in post-mitotic cell progeny [5]. Two classes of enzymes mediate histone acetylation, histone acetyltransferases (HATs) and histone deacetylases (HDACs), which, respectively, add or remove acetyl moieties to lysine residues in histone tails [6]. Epigenetic markers can be transient or maintained over long periods of time. Various external factors can trigger changes in epigenetics marks, such as diet, stress and cigarette smoking [7]. The effects of epigenetic marks are complex, but it is commonly accepted that DNA methylation suppresses, whilst histone acetylation promotes gene expression (although exceptions exist) [8,9]. Investigations into epigenetic aberrancies underlying COPD have shown differentially methylated sites in COPD leukocytes [10] and small airway epithelial cells [11]. Furthermore, we have shown histone acetylation aberrancies at the C-X-C chemokine ligand 8 (CXCL8) [12], collagen type XV alpha-1 (COL15A1) [13], and tenascin-C (TNC) [13] promoter regions unique to COPD. Thereby warranting further research to elucidate the epigenetic drivers of ASM aberrancies in COPD.

The nascent field of epigenetic therapeutics has shown promise in oncology, where the DNMT inhibitor (DNMTi), 5-azacytidine (trade name: Vidaza), obtained FDA approval for the treatment of myelodysplastic syndrome [14] and the HDAC inhibitor (HDACi) suberoylanilide hydroxamic acid (trade name: Vorinostat) obtained FDA approval for the treatment of cutaneous T-cell lymphoma [15]. Further, a phase I/II clinical trial that reversed gene silencing with a combination of DNMTi and HDACi treatment may prime patients with non-small cell lung cancer to respond better to subsequent chemotherapy, such as anti-PD1 monoclonal antibody nivolumab [16]. However, there remains a paucity of studies investigating how epigenetic therapeutics may be combined with current COPD treatment regimens. DNMTi and HDACi treatment non-selectively alter gene transcription [17], and HDACs can deacetylate lysine residues in many proteins [17], not just histones. Therefore, it is prudent to elucidate the effects of epigenetic inhibition on signaling proteins in the context of COPD before examining the use of these drugs in combination therapy.

This investigation offers a dataset derived from a selected cohort controlling for smoking history to reveal the differential effects of DNTMi 5-azacytidine (5-aza) and HDACi trichostatin-A (TSA) in COPD. We have placed this question in the context of the generation and release of CXCL8, a proinflammatory cytokine elevated in stimulated ASM cells from all smokers [18,19] and signaling protein abundance and phosphorylation.

## 2. Materials and Methods

### 2.1. Patient Cohort

All human lung tissues were obtained with written and informed consent from all volunteers or next of kin. Samples used in this investigation were approved by the Ethics Review Committee of the Southwest Sydney Area Health Service, Royal Prince Alfred Hospital and the University of Technology Sydney Ethics Committee (HREC #ETH16-0507; St Vincent’s Hospital HREC/15/SVH/351). Primary human airway smooth muscle cells were isolated from subjects either with COPD (forced expiratory volume in one second (FEV_1_)/forced vital capacity (FVC) < 70%) or without obstructive lung diseases. Those samples derived from non-obstructive lung disease were derived from the negative resected margins >5 mm from tumor boundary within resected tissues and defined as “normal adjacent tissue” (NAT). Patients were matched for smoking history and biological sex to control for epigenetic changes induced by these parameters. Patient demographics are provided in Table 1.

### 2.2. Airway Smooth Muscle Cell Isolation and Treatments

Primary airway smooth muscle cells were isolated from the right middle lobe from patients undergoing resection for thoracic malignancies (NAT) or lung transplantation, as previously described [13]. Briefly, the small airways (<2 mm diameter) were isolated from the right middle lobe. In resected tissue, ASM cells were obtained from regions deemed to be tumor-free following pathological examination. The ASM cells were selected using forceps and grown in a T25 cell-culture flask with Dulbecco’s Modified Eagles Medium (DMEM, #31600091, ThermoFisher Scientific, Waltham, MA, USA) supplemented with 10% fetal bovine serum (FBS; #16000044, ThermoFisher) and 1% antibiotic/antimycotic (#15240096, ThermoFisher Scientific) and buffered with 25 mM HEPES (#BIOHB0265, Astral Scientific, Taren Point, NSW 2229, Australia). Cells were expanded by a minimum of two passages into a T175 flask and were used in these experiments before passage six. The cells were incubated at 37 °C/5% CO_2_.

Considering the differing mechanisms of action between 5-aza and TSA, different time courses of treatment were used in our in vitro model. DNMT1i was induced with the addition of 5-aza (10 μM) (Cat#A2385, Sigma-Aldrich, St. Louis, MO, USA) dissolved in 0.05% (*v*/*v*) dimethyl sulphoxide (DMSO) (Cat#D2438, Sigma-Aldrich) directly to proliferating cells for 48 h and maintained in all media throughout the course of the experiment. Cells were brought to a G_0_ state through serum starvation for 24 h before treatment with ±TGF-β1 (10 ng/mL). HDACi was induced with TSA (100 nM) (Cat#T8552, Sigma-Aldrich) dissolved in DMSO during serum starvation for 24 h, then treated ± TGF-β1 (10 ng/mL).

### 2.3. ELISA

Cell-free supernatant was collected at 24 h post-TGF-β1 stimulation to quantify CXCL8 production using an enzyme-linked immunosorbent assay (ELISA) as previously described [20]. All samples from each donor were analyzed individually for all analyses.

### 2.4. Western Blot

Western blot analysis was completed as previously described [14]. Briefly, whole-cell protein lysates were collected using RIPA buffer. Total and phosphorylated (phospho) levels of signaling proteins involved in CXCL8 transcription—transcription factor NF-κB (Total: #8242; phospho: #93H1, Cell Signaling, Boston, MA, USA), and kinases p38 MAPK (total: #9212; phospho: #9211S, Cell Signaling), and JNK (total: #9258; phospho: #9251, Cell Signaling)—were quantified at 10, 20, and 30 min post-TGF-β1 stimulation using SDS-PAGE immunoblotting, as previously described [20]. Protein band densitometry was completed using the Carestream MI SE software (v5.0.2.30). Relative protein abundance was determined compared to GAPDH (#MAB374, Merk Millipore, Burlington, MA, USA). All samples from each donor were analyzed individually for all analyses. 

### 2.5. Statistical Analysis

Graphpad Prism version 10.0 was used to perform all figure generation and statistical analyses. All data were analyzed using a two-way analysis of variance (ANOVA). A post hoc Fisher’s least significant difference (LSD) correction for multiple comparisons was applied. Only *p*-values that were less than 0.05 were considered significant and are represented in the figures as an asterisk (*).

## 3. Results

### 3.1. TGF-β1 with TSA or 5-Aza Induces Greater CXCL8 Production from Non-COPD ASM

**Figure 1:**
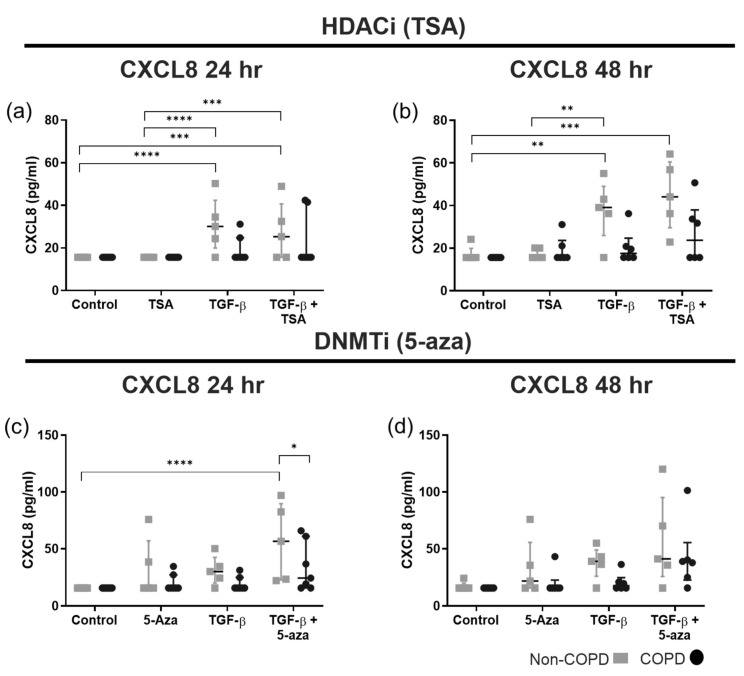
**CXCL8 production (pg/mL) from TGF-β1-stimulated (10 ng/mL) non-COPD (gray) and COPD (black) ASM cells.** Cells were pre-treated with either trichostatin A (TSA, 100 nM) (**a**,**b**) or 5-azacytidine (5-aza, 10 μM) (**c**,**d**) and incubated for 24 or 48 h. CXCL8 was determined in cell-free supernatant by ELISA. Data are presented as the median with the interquartile range and analyzed by two-way ANOVA with post hoc Fisher’s LSD test for multiple comparisons; *n* = 5–7. Statistical significance is represented by * *p* < 0.05, ** *p* < 0.01, *** *p* < 0.001 and **** *p* < 0.0001.

The 24 h HDACi model (Figure 1a) demonstrated that non-COPD ASM cells produced significantly higher levels of CXCL8 when stimulated with TGF-β1 ± TSA but not with TSA alone. These differences became more pronounced after 48 h of stimulation (Figure 1b). As a result, we postulate that the aberrance lies within the TGF-β1 pathway in a manner not abrogated by TSA. Alternatively, COPD-derived cells may carry a greater acetylome status, highlighting an inherent epigenetic alteration associated with the disease [21]. Our 24 h DNMTi in vitro model (Figure 1c) found that 5-aza alone failed to induce significant differences in COPD and non-COPD ASM CXCL8 production, but combined treatment with TGF-β1 with 5-aza caused significant upregulation of CXCL8 in non-COPD ASM compared to no-treatment control and COPD cells (Figure 1c). However, these differences disappear after 48 h of TGFβ-1 stimulation (Figure 1d). This indicates a catch-up or delayed response from COPD ASM compared to non-COPD in response to DNMTi. Our findings show that CXCL8 is differentially produced by COPD and non-COPD cells in a TGF-β1-dependent manner, with TSA not abrogating and 5-aza bolstering this effect.

### 3.2. TGF-β1 and 5-Aza Alter Total NF-κB Levels in Non-COPD ASM Cells

**Figure 2:**
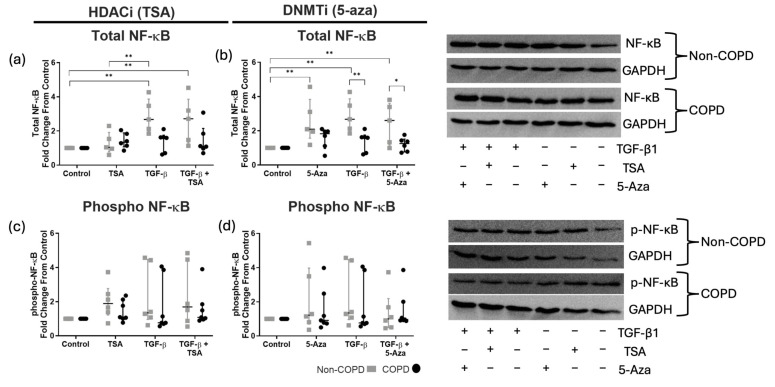
**Western blot quantification of total and phosphorylated NF**-κB **abundance in non-COPD (gray) and COPD (black) ASM cells.** Shown are total (**a**,**b**)/phosphorylated (**c**,**d**) NF-κB after 10 min TGF-β1 stimulation (10 ng/mL) in the presence and absence of trichostatin A (TSA, 100 nM) or 5-azacytidine (5-aza, 10 μM). Data are presented as median and the interquartile range and analyzed by two-way ANOVA with post hoc Fisher’s LSD test for multiple comparisons; *n* = 6–7. Statistical significance is represented as * *p* < 0.05 and ** *p* < 0.01.

Although we measured proteins within the CXCL8 signaling pathway at the 10, 20, and 30 min time points, we only included those time points where significant differences were recorded. Using the HDACi model, we found that total NF-κB abundance was significantly increased in non-COPD ASM cells stimulated with TGF-β1 ± TSA, compared to baseline and COPD ASM cells (Figure 2a). DNMTi increased the total NF-κB abundance significantly from non-COPD-derived ASM cells (Figure 2b). Both 5-aza treatment alone and combined with TGF-β1 increased NF-κB levels in non-COPD cells compared to COPD-derived cells. However, *p*-NF-κB levels were not different between non- and COPD-derived ASM cells in either the HDACi or DNMTi models (Figure 2c,d).

### 3.3. TGF-β1 Alters p38 MAPK Phosphorylation in Non-COPD-Derived ASM Cells Alone

**Figure 3:**
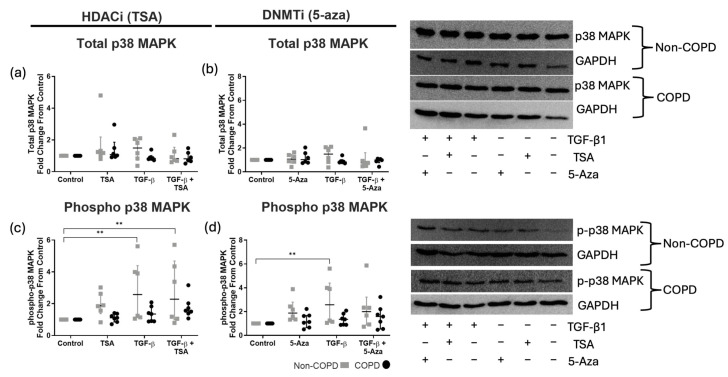
**Western blot quantification of total and phosphorylated p38 MAPK abundance in non-COPD (gray) and COPD (black) ASM cells.** Shown are total (**a**,**b**)/phosphorylated (**c**,**d**) p38 MAPK after 10 min TGF-β1 stimulation (10 ng/mL) in the presence and absence of trichostatin A (TSA, 100 nM) or 5-azacytidine (5-aza, 10 μM). Data are presented as median and the interquartile range and analyzed by two-way ANOVA with post hoc Fisher’s LSD test for multiple comparisons; *n* = 6–7. Statistical significance is represented as ** *p* < 0.01.

Both HDACi and DNMTi models demonstrated no change in total levels of p38 MAPK protein levels (Figure 3a,b). However, phosphorylated p38 MAPK levels were significantly increased in non-COPD-derived ASM cells stimulated with TGFβ1 ± TSA (Figure 3c). No effect of 5-aza was observed (Figure 3d). However, TGFβ1 treatment alone demonstrated a significant increase in p38 MAPK phosphorylation levels in only non-COPD-derived ASM cells (Figure 3c,d). No statistically significant changes in p38 total or phosphorylated proteins were reported from COPD-derived ASM cells.

### 3.4. TGF-β1 Combined with TSA or 5-Aza Alters JNK Phosphorylation in Non-COPD-Derived ASM Cells

**Figure 4:**
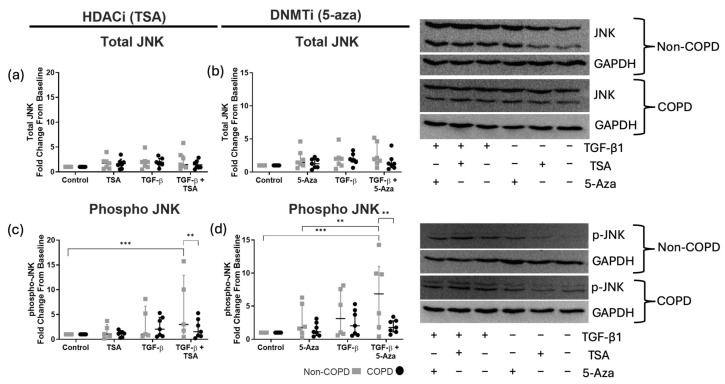
**Western blot quantification of total and phosphorylated JNK abundance in non-COPD (gray) and COPD (black) ASM cells.** Shown are total (**a**,**b**)/phosphorylated (**c**,**d**) JNK was measured after 20 min TGF-β1 stimulation (10 ng/mL) in the presence and absence of trichostatin A (TSA, 100 nM) or 5-azacytidine (5-aza, 10 μM). Data are presented as median and the interquartile range and analyzed by two-way ANOVA with post hoc Fisher’s LSD test for multiple comparisons; *n* = 6–7. Statistical significance is represented as ** *p* < 0.01 and *** *p* < 0.001.

Total JNK protein abundance was unchanged in both non- or COPD-derived ASM cells in both HDACi and DNMTi models (Figure 4a,b). Similarly, no change in JNK phosphorylation was reported for the treatment of TSA, 5-aza or TGF-β1 alone. Combined treatment of TGF-β1 + TSA induced a significant increase in JNK phosphorylation in only non-COPD-derived cells, compared to baseline and compared to stimulated COPD-derived cells (Figure 4c). Similarly, in the DNMTi model, co-stimulated non-COPD-derived ASM cells reported more JNK phosphorylation compared to baseline and co-stimulated ASM-derived cells (Figure 4d). This indicated a synergistic induction of increased JNK phosphorylation by TGF-β1 combined with either HDAC or DNMT inhibitors, which is specific to non-COPD-derived cells. No effect on JNK phosphorylation is observed for COPD-derived cells across all experimental conditions (Figure 4).

## 4. Discussion

The interrelationship between ASM, inflammation and small airway fibrosis in COPD is highly complex [22]. The action of the potent pleiotropic signaling molecules TGF-β1 and CXCL8 in the small airways is central to the early stages and progression of COPD. The current study has used airway smooth muscle cells from smokers with and without COPD to demonstrate that although CXCL8 is elevated in all smokers, the cytokine’s expression is differentially modulated by epigenetic inhibitors in COPD. Furthermore, we demonstrate that this aberrant production of CXCL8 is TGF-β1 dependent and signaling proteins in the CXCL8 cascade are differentially regulated in response to epigenetic inhibitors.

A foundational investigation of a similar phenomenon by Ito et al. [23] reported significantly reduced HDAC activity with COPD severity and compared to non-smoker controls. This finding, in part, explains our results, where we recognized limited effects of an HDAC inhibitor (TSA) in COPD-derived ASM cells compared to non-COPD controls. However, this publication, amongst others [24,25], highlight increased CXCL8 production in patients with COPD compared to controls. These studies investigated heterogeneous cell compartments of the body. As such, CXCL8 production from specific cellular compartments, such as the airway epithelium or immune cells, reveals contradictory results [26] with significant amounts of CXCL8 secreted by neutrophils and macrophages [25]. Further, studies report opposing expression patterns for the TGFβ-receptors (I and II) in relation to COPD status. For example, TGFβ-receptor expression is increased in the epithelium and alveolar macrophages of patients with COPD [27], whilst bronchial glands indicate reduced TGFβ-receptor expression [28]. These complex patterns of expression for both TGF-β and its receptor may be a key factor driving disease processes in COPD.

We previously showed that, fundamentally, TSA treatment increased CXCL8 production from cells [29], with HDAC inhibition linked to increased NF-κB activation [23]. Instead, we report reduced production of NF-κB protein in COPD compared to non-COPD airway smooth muscle cells. This change occurs in parallel with a distinct increase in JNK phosphorylation from non-COPD ASM with stimulation by TGFβ combined with both TSA and 5-aza. Combined, this understanding highlights the underlying cell-specific signaling mechanisms governing the inflammatory responses in COPD.

**Figure 5:**
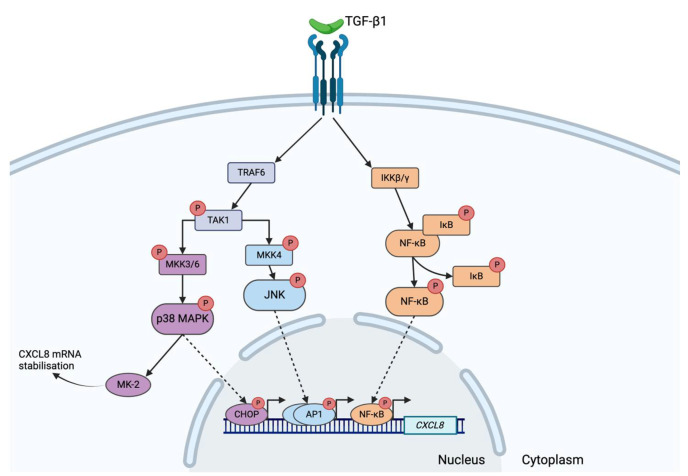
**Schematic of the TGF-β1 signaling cascade.** The straight solid arrows represent the direction of protein interactions and activation of signaling molecules. The dotted arrows represent signaling proteins translocating from the cytosol to the nucleus. The bent solid arrows represent the initiation of gene expression for CXCL8 due to activation of the respective transcription factor. The red circles containing a ‘P’ represent proteins that have been phosphorylated. *Figure generated using Biorender*.

The transcription factor JNK demonstrated increased activation in non-COPD ASM cells after TGF-β1 co-stimulation with TSA and 5-aza. JNK has been associated with CXCL8 and inflammatory responses from airway smooth muscle cells [30,31,32]. This reinforces a functional and specific role for this transcription factor in ASM cells. This becomes particularly important as we observe a lack of responsiveness of this transcription factor in COPD. This highlights a dysregulation of inflammatory responses in COPD post-epigenetic modulation. Therefore, fundamental acetylation and DNA methylation factors are absent in a disease state, potentially contributing to the poorly regulated immune response. The TGF-β1 intracellular signaling cascade is highly complex (summarized in Figure 5) and is still not completely understood.

The presented body of work is an initial step towards improving our foundational understanding of epigenetics in respiratory disease. Despite our selection of a curated patient cohort that is matched for sex and smoking history, other factors require follow-up investigations. Our investigation leverages normal adjacent tissue samples to function as substitutes for the scarce resource of “healthy” samples. We acknowledge that cancer-adjacent tissues can carry altered epigenetic profiles [33]. This is an ongoing challenge, which is constantly evolving via studies unraveling the complexities of DNAm modifications in normal adjacent tissues [34,35]. The use of NAT controls in the current study functions to illustrate an altered epigenetic profile that is distinct from COPD ASM cells. Further studies are required to appropriately resolve the consequences of these alterations in the broader context of non-diseased vs. diseased states.

Aging does contribute to changes in DNA methylation across the entire genome [36]. Our cohort is limited by the relatively older age of our non-COPD subset, reducing the ability to broadly interpret and apply the findings across the general population. As such, the inclusion of a broader age range in future studies would provide greater insight into the full spectrum of epigenetic changes associated with younger or older age brackets in both COPD and non-COPD individuals. Importantly, cigarette smoking, as a significant strong epigenetic modifier and key component of COPD development [37,38,39], is controlled for in this study. In addition, COPD demonstrates sex differences [40]. An expansion of the cohort to generate sufficient power to conduct sex-stratified analysis will likely uncover pertinent sexually dimorphic patterns of DNA methylation in response to 5-aza and TSA. Future investigations using these epigenetic inhibitors would benefit by combining both 5-aza and TSA to block DNA methylation and histone acetylation simultaneously, which would certainly uncover valuable insights regarding the relationship/overlap between these processes. Similarly, including pathway inhibitors for 5-aza and TSA would help confirm the specificity of their effects on pathway activation in the context of COPD. In addition, a deeper, multi-omics characterization of the consequences of the epigenetic changes could be achieved via ATAC-/RNA-sequencing. Determining the functional consequences of changes or differences in epigenetic profiles between patient groups is a foundational step to holistically understanding such complex mechanisms. These techniques used in combination may reveal novel gene networks that are dysregulated, in addition to CXCL8, that can function as biomarkers in COPD. Such future analyses would effectively build upon the current body of work and greatly propel our understanding of the role of epigenetic mechanisms in respiratory disease.

## 5. Conclusions

We present that TGF-β1-induced CXCL8 production from ASM cells is reduced in COPD compared to non-COPD. This is particularly caused when epigenetic inhibitors are used in combination with this altered response associated with differential activation of transcription signaling protein, namely JNK. Our results, along with other publications, indicate that future studies should consider the epigenetic regulation (as impacted by these drugs: TSA and 5-aza) of NF-κB [41] and kinases upstream from p38 MAPK and JNK [42,43] responsible for their phosphorylation, such as those within the MAP2K or MAP3K families. However, analysis of this phenomenon in the context of COPD is lacking. We encourage further research on the extent to which signaling pathways are affected by the epigenetic profile of COPD. These results present a step towards understanding how mesenchymal cell epigenetics underpin aberrant inflammatory pathways in COPD.

## Figures and Tables

**Table 1:** Patient demographic information. NAT = normal adjacent tissue; FEV1_1_ = forced expiratory volume in one second; FVC = forced vital capacity.

Details	COPD	Non-COPD
**All Patients**	*n* = 7	*n* = 7
**Age Range (Mean ± SD)**	44–60 (54.8 ± 6.2)	60–75 (66.5 ± 6.1)
**Diagnosis**	COPD	Normal Adjacent Tissue
**Smoking History**	>40 pack years	>40 pack years
**Gender (M:F)**	4:3	4:3
**FEV_1_/FVC**	<0.4	>0.80
**Surgery**	Transplant	Resection

## Data Availability

The datasets generated and/or analyzed during the study are available from the corresponding author upon reasonable request.
